# Supplementary material for: Association of aflatoxin B1 levels with mean CD4 cell count and uptake of ART among HIV infected patients: A prospective study
Source: PLoS One. 2022 Jan 27;17(1):e0260873. doi: 10.1371/journal.pone.0260873 (PMC8794094; doi:10.1371/journal.pone.0260873)
Supplement: S1 Table — (DOCX) [file pone.0260873.s001.docx]

| **Supplemental Table: Multivariable adjusted models of mean CD4 estimates over the study period (with aflatoxin at baseline as continuous variable)** | | | | | | |
| --- | --- | --- | --- | --- | --- | --- |
|  | **Model 1** | | **Model 2** | | **Model 3** | |
|  | **Beta estimates** | **P value** | **Beta estimates** | **P value** | **Beta estimates** | **P value** |
| **AF-ALB pg/mg (baseline)** | -2.3 | **0.024** | -1.6 | 0.251 | -1.6 | 0.215 |
| **On ART** |  | **<0.001** |  | **<0.001** |  | **0.001** |
| No | 149.0 |  | 126.0 |  | 120.5 |  |
| Yes | Ref |  | Ref |  | Ref |  |
| **Gender** |  | **0.037** |  | **0.028** |  | **0.030** |
| Female | 85.1 |  | 103.2 |  | 104.8 |  |
| Male | Ref |  | Ref |  | Ref |  |
| **Age** |  | 0.627 |  | 0.944 |  | 0.897 |
| 18-29 | 21.9 |  | -21.9 |  | -21.5 |  |
| 30-39 | 8.0 |  | -31.2 |  | -28.3 |  |
| 40 and above | Ref |  | Ref |  | Ref |  |
| **Socioeconomic status** |  | **0.030** |  | **0.032** |  | **0.017** |
| Low | 84.4 |  | 78.0 |  | 92.1 |  |
| Middle | 56.2 |  | 19.3 |  | 30.3 |  |
| High | Ref |  | Ref |  | Ref |  |
| **Season** |  | 0.115 |  | 0.102 |  | 0.210 |
| Dry | -48.2 |  | -36.4 |  | -36.2 |  |
| Rainy | Ref |  | Ref |  | Ref |  |
| ^1^Adjusted for age, gender, SES rank, art status, season, aflatoxin level at baseline, knowledge of HIV-positive status  ^2^Adjust for model 1 variables + alcohol consumption + food consumption patterns  ^3^Adjust for model 2 variables + health status + viral load + Hepatitis B status; ART = antiretroviral therapy | | | | | | |
