# Supplementary material for: Association of aflatoxin B1 levels with mean CD4 cell count and uptake of ART among HIV infected patients: A prospective study
Source: PLoS One. 2022 Jan 27;17(1):e0260873. doi: 10.1371/journal.pone.0260873 (PMC8794094; doi:10.1371/journal.pone.0260873)
Supplement: S1 File — (PDF) [file pone.0260873.s002.pdf]

BASELINE QUESTIONNAIRE
AFLATOXIN AND HEALTH STATUS IN HIV DISEASE

Participant ID ____________________ Date _______________

DEMOGRAPHIC AND SOCIO-ECONOMIC INFORMATION

1. Ethnic Group (Please select only one group)
   - ☐ Group 1: Akan
   - ☐ Group 2: Gruma, Busanga, Bimoba and Kusasi
   - ☐ Group 3: Dagbani, Basare/Basali, Gonja and Konkomba
   - ☐ Group 4: Moshi, Hausa, Waa, Kokama, Busani, Gungui and Zugu
   - ☐ Group 5: Ewe, Dagati, Sisala, Wala, Grussi, Frafra and unknown

2. Sex of participant ☐ male ☐ female
3. Age _____ (Date of birth /___ /___ /___/ (day/month/year)
4. Town/village ______________________________
5. Age of the head of household (in years) /___/___/
6. Marital status ☐married ☐single ☐divorced ☐separated ☐window(er) ☐cohabiting
7. Sex of head of household ☐ male, ☐ female
8. Level of education (give the highest level) Number of years

☐ None	
☐ Primary	_________(years)
☐ JSS	_________(years)
☐ MSLC	_________(years)
☐ Secondary	_________(years)
☐ College	_________(years)
☐ Technical	_________(years)
☐ University	_________(years)
☐ Other (Specify) ____________________________	

9. Do you work? ☐ Yes ☐ No
   ☐ Other: Specify _______________________ _______

10. If yes to question number 9, what is your occupation?
    - ☐ Farmer
    - ☐ Trader

- ☐ Laborer
- ☐ Technical specialty (Motor mechanic, electrician)
- ☐ Professional (Teacher, engineer)
- ☐ Food/Products vendor
- ☐ Other _________________

11. State monthly income from your work? (Cedis)

☐ 50-100 (Cedis/month) ☐101-150 ☐151-200 ☐ 201-250 ☐ >250 specify__________

12. State monthly income from sources other than work?

Amount: _______(Cedis/month) ; Source: ________________

13. Number of individuals in your household. ____________
14. Number of individuals in your household who work. __________
15. Estimated total household income (All sources in Cedis/Month) ________
16. How would you describe where you live?

☐ Own home ☐ Rent ☐ Live with family/friend
☐ Student housing ☐ Government housing ☐ Temporary housing ☐ Other

17. Does the place you live have the following:

Plumbing ☐ Y ☐ N
Piped water ☐ Y ☐ N
Electric lighting ☐ Y ☐ N

18. Of what primary material is your house/living quarters constructed?

☐ Wood ☐ Cement ☐ Brick ☐ Other

19. How many people sleep in each room of your house/living quarters? ___________
20. With what religious group are you affiliated?

☐ Christian (Protestant) ☐ Muslim ☐ Traditional
☐ none ☐Other____________

FAMILY DEMOGRAPHIC INFORMATION

21. Number of individuals living in the household (including yourself) ________
22. Number of children 9 years and under in the household _________
23. Number of children between 10 and 15 years________
24. Number of children attending primary school ________
25. Number of children attending secondary school __________
26. Number of children attending tertiary/college level ________

PARTICIPANT'S DRINKING / SMOKING HISTORY

27. Do you drink alcoholic beverages?

☐ Yes ☐ No

28. If yes, specify beverage(s) and number of glasses/bottles per week.

Beverage(s)	Number of glasses/bottles/week
☐ Wine	____________________
☐ Beer	____________________
☐ Liquor	___________________
☐ Locally distilled (e.g. Pito, akpeteshie)__________	

29. Do you smoke cigarettes?

☐ Yes ☐ No

30. If yes, type? ☐ Store bought ☐ Hand rolled

31. If yes, number _____ per day.

32. Do you drink coffee?

☐ Yes ☐ No

33. If yes, number of cups_____ per day.

PARTICIPANT'S HEALTH FACTORS

34. How would you describe your health during the last 6 months?

☐ Very healthy ☐ Average health ☐ Poor health

35. How often do you get sick each month?

☐ Never ☐ 1-2 times ☐ 3-4 times
☐ 5-6 times ☐ 7-8 times ☐ 9-10 times ☐ More than 10 times per month

36. When was the last time you were sick? ______ days/weeks ago
How long were you ill? _______ (days/weeks)

37. Have you had any of the following conditions?

	**Condition**	**Have the following conditions**		**Month/Year**	**Hospitalized** 1= Yes 2=No	**No. of Days Hospitalized**
		**Yes**	**No**			
A.	Yellowing of the skin (e.g., palm of your hands)					
B.	Yellowing of the whites of the eyes					
C.	Dark brown color of urine					
D.	Yellowing of the mouth					
E.	Vomiting					
F.	Abdomen pain/distended abdomen					
G.	Pale /gray stool					
H.	Fatigue/ feelings of extreme tiredness					

38. How many times have you been hospitalized in your life? ______

39. When was the last time you were hospitalized? _____ days ago

_____weeks ago

_____months ago

_____years ago

40. Regarding your last hospitalization, how long did you stay in the hospital?_______ Days/Weeks

41. How long were you sick before going to the hospital? ________Days/Weeks

42. Do you believe that your illness or signs/symptoms were related to eating any of the following?

Check all that apply.

☐ Peanuts ☐ Peanut products ☐Maize ☐ Maize Products ☐Other grain or food, specify ____________

43. What was the illness diagnosed by the doctor? ____________________

FOOD ACQUISITION, CONSUMPTION, AND PRACTICES

44. What proportion of your household food do you and your family grow/produce?

☐None ☐ <20% ☐ 20- 50% ☐ 51-70% ☐ >70

45. What proportion of food do you and your family buy?

☐ <20% ☐ 20- 50% ☐ 51-70% ☐ >70

46. Select from the list below the frequency of foods you consume weekly, whether you sort the food, and the place of storage before consumption

**Food type**	**Frequency of eating times per week**<br>**1=never**<br>**2=one time or less**<br>**3=2-3 times**<br>**4=everyday**	**Sort**<br>**1=all the time**<br>**2=some time**<br>**3=rarely**	**Place of storage before consumption**<br>**1=kitchen**<br>**2=storage house**<br>**3=house**<br>**4=other**
**Groundnut**			
Groundnut paste<br>Groundunt soup<br>Groundnut sauce			
**Maize**			
Maize Products<br>Kenkey<br>Banku<br>Apeprensa<br>Asana			
**Millet**			
Millet products			
**Sorghum**			
Sorghum products			
**Boiled Cassava**			

**Cassava products**<br>Gari<br>Cassava fufu			
Other			

47. What proportion of maize/maize products that you ate in the last month was stored before use?

☐ < 25% ☐ < 50% ☐ > 50% ☐ 100%

48. How many months was the maize stored before consumption?

☐ 0-2 m ☐ 3-5 m ☐ 6-9 m ☐ 10-12 m ☐ >12 m

49. Can you identify spoilage in any of the food items that you consume?

☐ Yes ☐ No.

50. What do you do with food items you consider to be spoilt daily?

☐ Discard always ☐ Discard sometimes ☐ Eat ☐ Feed to Animals

51. How many kg of your maize is usually spoilt?

☐ < 0.5 kg ☐ < 1.0 kg ☐ > 1.0 kg ☐ > 2.0 kg ☐ >5 kg

Awareness of aflatoxin

52. Have you ever heard of aflatoxin before? ☐ Yes ☐ No ☐ Don't know
53. Do you know whether it causes any sickness? ☐ Yes ☐ No ☐ Don't know
54. Do you know with which food it is most associated? ☐ Yes ☐ No ☐ Don't know
55. Are you aware of aflatoxin contamination of groundnuts? ☐ Yes ☐ No ☐ Don't know
56. Are you aware of aflatoxin contamination of maize? ☐ Yes ☐ No ☐ Don't know
57. Are you aware of the health effects of aflatoxins in humans? ☐ Yes ☐ No ☐ Don't know
58. Are you aware of the health effects of aflatoxins in animals? ☐ Yes ☐ No ☐ Don't know
59. Do you discuss aflatoxins with your family or neighbors? ☐ Yes ☐ No ☐ Don't know
60. Discolored nuts/grains are not harmful when eaten. ☐ Yes ☐ No ☐ Don't know
61. Damaged and broken nuts/grains do not spoil other healthy nuts/grains in storage. ☐ Yes ☐ No ☐ Don't know

<u>HIV/AIDS/STD INFORMATION</u>

62. How do you believe you contracted HIV?

☐ Sex with men
☐ Sex with women
☐ Sex with both men and women
☐ Intravenous drug use
☐ By contact with person other than sex
☐ Sharing sharps (blades, needles etc.)
☐ Don't know

63. Have you had any sexually transmitted diseases in the last year? ☐ Yes ☐ No
If yes, do you know which?
☐ Chlamydia ☐ Gonorrhea ☐ Syphilis ☐ Chancroid ☐ Hepatitis B/C ☐ Herpes
☐ Other, specify__________

64. How long have you known that you are HIV+?
Years______________ Months________________

Date of first HIV test----MM/YY /___/___/

65. Are you currently experiencing any of the following symptoms?

☐ Diarrhea
☐ Difficulty swallowing
☐ Persistent headaches
☐ Oral plaques/rashes
☐ Vomiting
☐ Fever
☐ Persistent cough
☐ Skin rash
☐ Sexually transmitted infections
☐ Weight loss (>10%)
☐ Visual changes
☐ Other, specify_______________

66. Have you ever been hospitalized for an illness related to your HIV infection?
☐ Yes ☐ No ☐ Don't know

67. If yes for question 80, please specify the illness:

☐ Chronic diarrhea
☐ Skin rash
☐ Oral plaques/rashes
☐ Pneumonia
☐ Sexually transmitted infections
☐ Severe weight loss (>10%)
☐ Visual changes
☐ Other respiratory infections
☐ Other, specify ______________________

68. How many times have you been hospitalized for HIV-related illnesses over the past 12 months?
Total number of hospitalizations __________

69. Do you take any local medication(s)?
☐ Yes ☐ No
If Yes, which and how often Name_______________________ _______/week
Name ___ ____________________ ______ /week
Name ________________________ _______/week

70. Do you take any vitamins or other supplement?
☐ Yes ☐ No
If Yes, which and how often Name________________ ______ Dose/per day
Name ________________ ______ Dose/per day
Name ________________ _______Dose/per day
